# A Phase II study of gemcitabine and cisplatin in chemotherapy-naive, unresectable gall bladder cancer

**DOI:** 10.1038/sj.bjc.6601736

**Published:** 2004-03-16

**Authors:** D C Doval, J S Sekhon, S K Gupta, J Fuloria, V K Shukla, S Gupta, B S Awasthy

**Affiliations:** 1 Rajiv Gandhi Cancer Institute & Research Center, Delhi, India; 2 Dayanand Medical College & Hospital, Ludhiana, India; 3 Dharamshila Cancer hospital & Research Center, New Delhi, India; 4 Ochsner Clinic Foundation, New Orleans, USA; 5 Institute of Medical Sciences, Banaras Hindu University, Varanasi, India; 6 Eli Lilly and Company Pvt. Ltd, India

**Keywords:** gall bladder cancer, gemcitabine, cisplatin

## Abstract

The primary objective of this study was to determine the response rates of the gemcitabine and cisplatin combination in unresectable gall bladder cancer patients. The secondary objectives were to evaluate the toxicity, time to progressive disease, and overall survival. Chemonaïve patients with histologically proven, unresectable bidimensionally measurable gall bladder cancer were enrolled into this study. All patients were required to have a Zubrod's performance status ⩽2, no prior radiotherapy, and adequate major organ function. Patients received gemcitabine (1000 mg m^−2^ intravenously over 30–60 min) on days 1 and 8, and cisplatin (70 mg m^−2^ intravenously over 2 h) on day 1, every 21 days. Response assessment was done by a CT scan after every other cycle of chemotherapy. In all, 30 patients were eligible for efficacy and toxicity analysis. There were four (13.3%) complete responders, seven (23.3%) partial responders, and seven (23.3%) with stable disease, with four (13.2%) patients showing disease progression. The median time to progression was 18 weeks (95% confidence interval (CI) 14–24 weeks), and the median duration of response was 13.5 weeks (range 5.5–104 weeks). The median overall survival was 20 weeks (95% CI 14–31 weeks), with 1-year survival rate of 18.6%. WHO grade 3 or 4 anaemia was seen in seven (23.3%) and four (13.3%) patients, respectively. Five (16.6%) patients each experienced grade 3 or 4 neutropenia, and grade 3 or 4 thrombocytopenia was seen in three (10%) and two (6.6%) patients, respectively. The present study shows that gemcitabine/cisplatin combination is well tolerated and active in advanced unresectable gall bladder cancer.

Adenocarcinoma of the gall bladder and biliary ducts accounts for approximately 4% of all malignant neoplasms of the gastro-intestinal tract (Greenlee *et al*, 2001), and remains a major challenge to surgical, medical, and radiation oncologists. For a small percentage of patients who present with resectable disease, the treatment of choice is surgical resection. However, the general outcome remains disappointing even in patients undergoing aggressive surgery (De Groen *et al*, 1999).

Most patients with gall bladder cancer present with invasive, inoperable disease. Chemotherapeutic agents including 5-fluorouracil (5FU), mitomycin C, cisplatin, methotrexate, etoposide, and doxorubicin have been tried alone, and in combination, for this patient group. Partial responses lasting from weeks to several months have been observed only in about 10–20% of the cases, and the median survival for patients with gall bladder cancer is dismal at around 4 months (Verderame *et al*, 2000). Chemoimmunotherapy has shown encouraging results (Murakami *et al*, 1998; Hasegawa *et al*, 1999), but the data are limited to a few case reports only. Similarly, isolated reports of intra-arterial chemotherapy (Hara *et al*, 2000) and intralesional therapy (Makower *et al*, 2003) have been published. The poor therapeutic results, along with small sample size in the trials, preclude the support of any particular chemotherapeutic regimen for unresectable disease. Therefore, newer, more effective treatment strategies must be evaluated.

Gemcitabine is a pyrimidine analogue of deoxycytidine and has shown strong antitumour activity in a variety of solid tumours. Cisplatin is a stable platinum complex, which is converted into a reactive electrophile at lower chloride concentrations and binds covalently to DNA, resulting in apoptosis. Cisplatin has synergistic activity with gemcitabine (Peters *et al*, 1995).

Additionally, the gall bladder shares a common embryonic origin with exocrine pancreas, where gemcitabine has shown antineoplastic effectiveness (Moore, 1996). These factors prompted us to plan a phase II trial using a combination of gemcitabine and cisplatin in the treatment of chemotherapy-naive patients with unresectable gall bladder cancer. The primary objective of this study was to determine the response rate. The secondary objectives were to evaluate the toxicity, time to progressive disease (PD), and the overall survival for this combination.

## PATIENTS AND METHODS

### Eligibility criteria

Chemonaive patients, with histologically proven unresectable bi-dimensionally measurable gall bladder cancer on a CT scan or X-ray, were entered into this study. Patients >18 years of age; Zubrod's performance status ⩽2; ANC>1500 mm^−3^; platelets>100 000 mm^−3^; Hb>9 g%; child-bearing potential terminated by surgery, radiation, menopause, or attenuated by the use of an approved contraceptive method; and with an estimated life expectancy of at least 12 weeks were eligible. Patients who had received prior radiotherapy were eligible, provided that the irradiated area was not the only source of measurable disease and the radiation was completed at least 3 weeks prior to enrolment into the study. Patients were excluded if they were pregnant or breastfeeding, had a history of previous carcinoma in the last 5 years, and ALT/AST/transaminases >3 × ULN (in case of liver metastasis ALT/AST >5 × ULN). The study was conducted after obtaining approval from the Ethical Review Board of each participating institution, as well as written informed consent from each patient.

### Treatment plan

Patients received 1000 mg m^−2^ of gemcitabine in 100 ml saline via intravenous infusion over 30–60 min on days 1 and 8 of a 21-day cycle, in an outpatient clinic. Cisplatin, 70 mg m^−2^, diluted in 500 ml of 0.9% normal saline, was given intravenously on day 1, after completing the gemcitabine dose. Cisplatin dose was also preceded by pre-hydration and electrolyte supplementation as per institutional practice, or the manufacturer's recommendation. The cycle was repeated every 3 weeks for a maximum of six cycles, unless there was prior evidence of PD.

Drug doses were modified within a cycle based on white blood cells (WBC), platelet count, and clinical assessment of nonhaematologic toxicities carried out a day prior to chemotherapy. If the ANC was between 1000 and 1500 mm^−3^, and/or platelet count was between 75 000 and 100 000 mm^−3^, gemcitabine was administered at 75% of the full dose. If the ANC count was between 500 and 999 mm^−3^, and/or the platelet count was between 50 000 and 74 999 mm^−3^, gemcitabine was administered at 50% of the full dose. Gemcitabine was withheld if the ANC count was <500 mm^−3^, and/or the platelet count was <50 000 mm^−3^. Doses held for toxicity within a cycle were not to be given at a later time. Prior to starting a new cycle, a value of ANC>1500 mm^−3^ and platelets>100 000 mm^−3^ was required. In the event of neutropenia or thrombocytopenia the cycle was delayed, and CBC was repeated twice weekly until this value was reached. Prior to start of a new cycle, for serum creatinine levels between 1.6 and 2 mg dl^−1^, the cisplatin dose was decreased by 50%, and for levels >2.0 mg dl^−1^ cisplatin was to be withheld. If the value was higher than that mentioned above, creatinine was repeated weekly and the cycle was delayed until the value dropped to <2 mg dl^−1^. If cisplatin was held for any length of time (1–4 weeks), re-treatment was done at 50% of the full dose. If the criteria for re-treatment were not met within 4 weeks, the patient was to be taken off protocol. Based on the physician's discretion, a dose was either reduced or withheld for any nonhaematologic and nonrenal WHO grade 3 or 4 toxicity (except nausea/vomiting and alopecia).

Doses held due to toxicity or missed within a cycle were not to be given at a later time, and any patients who could not receive drug for 4 weeks were discontinued from the study. Chemotherapy was to be discontinued for PD, clinical deterioration, at the patient's request, due to pregnancy or medical judgment by treating physician, or for patients who received six cycles of chemotherapy.

### Baseline and treatment assessments

Within 2 weeks prior to starting therapy, patients were assessed by a detailed medical history, physical examination, chest X-ray, and CT scan of the abdomen. Prior to every new cycle of therapy, all patients were assessed with a limited medical history, physical examination, including weight and performance status, complete blood count, and renal and hepatic function tests. CT scan of the abdomen and chest X-ray were done every other cycle for response assessment. In addition, a complete blood count was carried out prior to giving the day 8 dose of gemcitabine.

All patients who received at least one cycle of chemotherapy with gemcitabine and cisplatin were eligible for efficacy and toxicity analysis. A complete response (CR) was defined as the disappearance of all known disease determined by two observations not less than 3 weeks apart. A PR was defined as at least a 50% decrease in measurable disease by two observations not less than 3 weeks apart, and no evidence of any new lesions or progression of any existing lesions. An inability to demonstrate a 50% decrease in tumour size or a 25% increase in the size of one or more lesions, as well as no new lesions, defined SD. A 25% increase in the size of one or more measurable lesions, or the appearance of any new lesions, defined PD.

The duration of response was calculated as the duration between the first documented response (CR/PR/SD) and PD or death, whichever is earlier. Survival was measured from the administration of the first dose until the date of death. Time to progression was defined as the time of administration of first dose of chemotherapy to the date of PD or death, whichever occurred earlier. Toxicity was recorded as per WHO criteria.

### Statistical analysis

The primary end point of this study was response rate. The width of the resultant confidence intervals (CIs) for parameters to be estimated were constructed with a significance level of 0.05, that is, a 95% CI. The Kaplan–Meier survival analysis method was used to estimate the median survival time for overall survival, as well as time to progression, and estimates were provided with 95% CIs. Statistical analysis was performed using SAS 8.02 (SAS Institute Inc.). Wilcoxon signed rank test was used to analyse the statistical significance of change in the grades of ZPS status from cycle 1 to cycle 3, and cycle 1 to cycle 6. Log rank test was used to compare the differences among the patients grouped based upon relative dose intensity (RDI) with median RDI as cutoff. Spearman rank correlation was performed to analyse the correlation between RDI and overall response for both the drugs. Cox proportional hazard model was used to analyse the effect of covariates on overall survival. A final analysis was based on all follow-up information received until August 2, 2002.

## RESULTS

### Patient characteristics

From February to August 2000, 30 patients from four different institutions were enrolled into the study, and all were evaluable for efficacy and toxicity analysis. The ratio of male to female patients was 8 : 22. Patient characteristics are enumerated in Table 1

**Table 1:** Patient characteristics (*N*=30)

**Sex**	
Male	8 (27%)
Female	22 (73%)
	
Median age (range)	53.5 years (21–75)
	
*Zubrod's performance status*	
1	22 (73%)
2	8 (27%)
	
*Method of diagnosis*	
Aspirate	21 (66.6%)
Biopsy	1 (3.3%)
Excision	9 (30%)
	
*Disease status*	
Metastatic	20 (66.6%)
Recurrent	7 (23.3%)
Locally advanced	3 (10%)
	
*Prior radiation*	2 (6.6 %)

**Figure 1:**
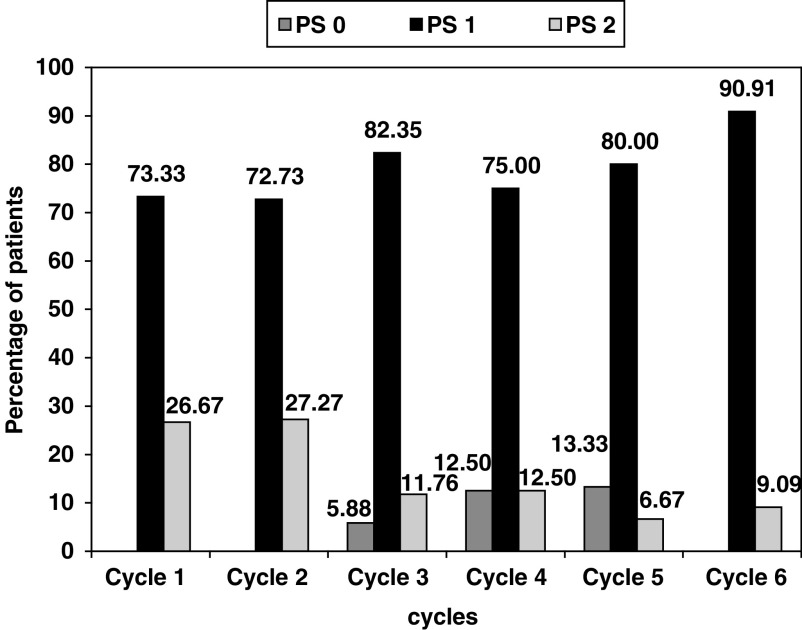
Changes in Zubrod's performance status across the cycle.

. In all, 22 (73.0%) patients had a Zubrod's performance status of 1, and 20 (66.6%) patients had metastatic disease. There was no significant change seen in the Zubrod's performance status compared from baseline to cycle 3 (*P*=0.62) or cycle 6 (*P*=1), as shown in Figure 1

**Figure 2:**
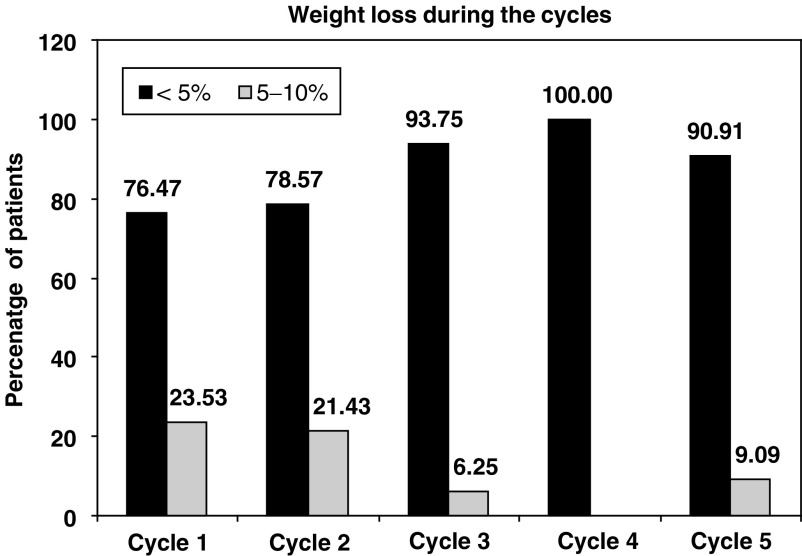
Changes in weight across the cycles.

. No grade>1 weight loss was observed during therapy (Figure 2

).

### Dose administration

A total of 110 cycles of chemotherapy were administered. Median number of cycles given was 4.5 (range 1–6). In all, 16 (53%) patients completed at least four cycles of therapy. The mean cumulative doses of cisplatin and gemcitabine received were 256 (±154) and 6934 (±4510) mg, respectively. The median RDIs of cisplatin and gemcitabine were 99 and 95%, respectively.

Of the 110 cycles administered in 30 patients, 25 cycles (24%) were delayed by a mean number of 3.5 days. Only five of these were delayed due to medical reasons, the remainder was due to social reasons. The day-8 dose of gemcitabine was omitted in eight cycles (7%) in six patients, and reduced in six cycles (5%) in five patients, with a 50% reduction in two cycles and a 75% reduction in four cycles. Cisplatin dose was reduced in one cycle due to nephrotoxicity.

### Response and time-to-event measures

**Figure 3:**
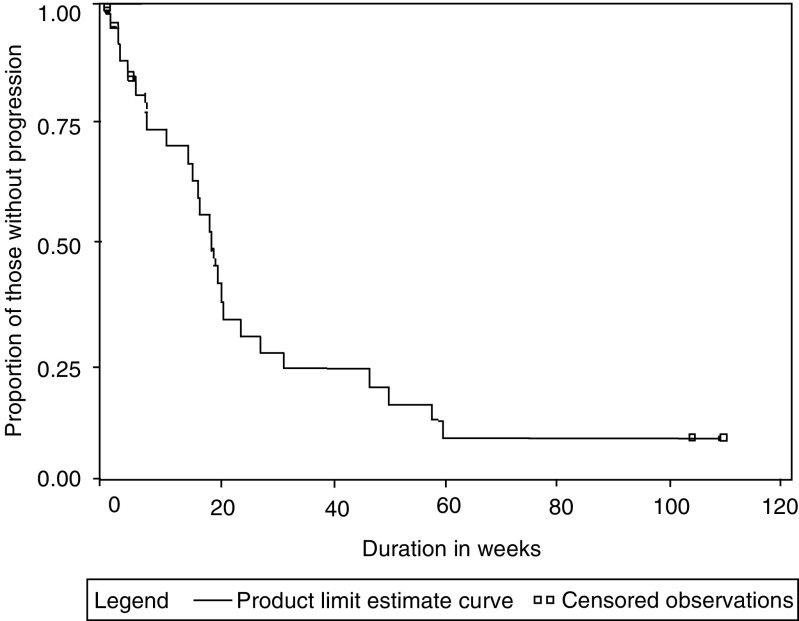
Kaplan Meier survival analysis for time to PD.

**Figure 4:**
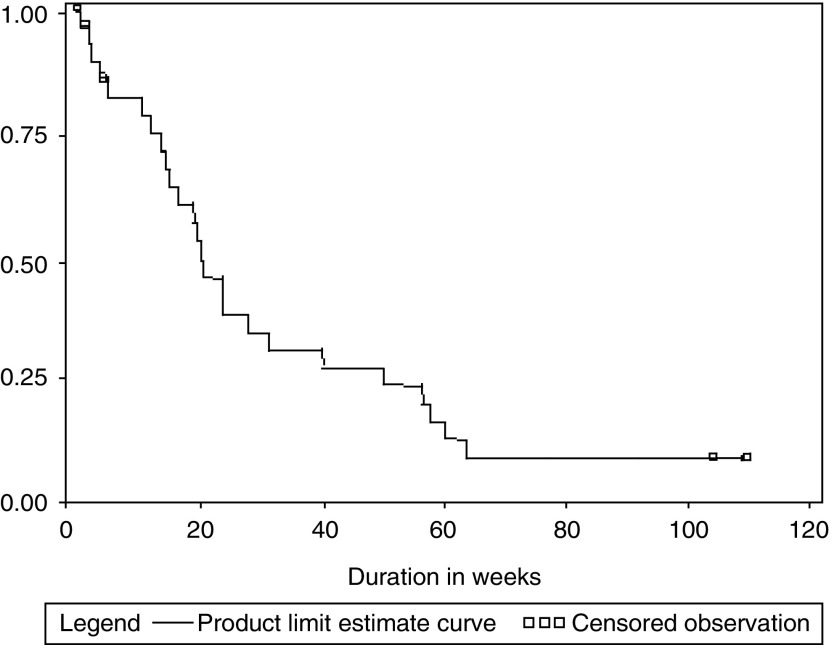
Kaplan Meier curve for overall survival of patients.

Four patients achieved a CR (13.3%), seven patients achieved PR (23.3%), seven patients had SD (23.3%), and four patients showed PD (13.2%). Response could not be assessed in eight (27%) patients. Of these eight patients, one withdrew consent, one was discontinued as per the physician, and three were lost to follow-up. These three patients failed to turn up after the first cycle of chemotherapy, and repeated attempts to contact them were not successful. There were three deaths before evaluation of response, two of which were thought to be treatment related (septicaemia and hepatic encephalopathy), and one was due to an unrelated cause (sudden cardiac arrest). This patient was a known diabetic for 8 years on irregular treatment, but did not have any known cardiac disease. In the opinion of the treating investigator (JS Sekhon), this event was not related to the therapy. The median TTP was 18 weeks (95% CI; 14–24 weeks; Figure 3

). The median overall survival was 20 weeks (95% CI 14–31 weeks), and 1-year overall survival was 18.6% (Figure 4

). The median duration of response (CR/PR/SD) was 13.5 weeks (range 5.5–104 weeks). The median duration of CR in four patients was 58 weeks (range 8–103 weeks). The median duration of PR was 20 weeks (range 15–60 weeks).

The number of chemotherapy cycles given was a significant prognostic factor affecting overall survival (Table 2

**Table 2:** Survival analysis for individual covariates

**Covariate**	**Stratification**	**Estimated median (weeks)**	**Test of equality (*P*-value for log rank test)**
Age	Less than 50	27.857	0.0517
	More than 50	19.429	
Zubrod's performance status	Grade 0,1	27.857	0.0211
	Grade 2,3,4	14.429	
No of cycles	Less than 3	10.429	<0.0001
	More than 3	35.643	
Sex	Male	18.214	0.3857
	Female	20.286	

). Cox proportional regression analysis indicates a significant effect of >94% RDI gemcitabine (*P*=0.0037), and more than four cycles of CT given (*P*=0.0031) on the overall survival of these patients (Table 3

**Table 3:** Cox proportional hazard regression with covariates

**Variable**	**Standard error**	***χ*^2^**	**Pr>*χ*^2^**	**Hazard ratio**
Gem RDI >94%	0.54465	8.4341	0.0037	0.206
Age >50 years	0.48050	0.3515	0.5533	1.330
Sex F	0.50615	0.0157	0.9003	0.939
Zubrod's PS ⩾2	0.66478	0.0893	0.7650	0.820
Cycles >3	0.66587	8.7275	0.0031	0.140

gem=gemcitabine, Pr=probability, RDI=relative dose intensity, PS=performance status.

).

### Toxicity

The grade 3 and 4 toxicities encountered are listed in Table 4

**Table 4:** WHO grade (G) 3/4 toxicity

	***N*=30 patients**		***N*=110 cycles**	
**Parameters**	**Grade 3 (%)**	**Grade 4 (%)**	**Grade 3 (%)**	**Grade 4 (%)**
Anaemia	7 (23)	4 (13)	12 (11)	5 (4)
Leukopenia	3 (10)	2 (7)	9 (8)	2 (1)
Granulocytopenia	5 (17)	5 (17)	9 (8)	7 (6)
Thrombocytopenia	3 (10)	2 (7)	5 (4)	2 (1)
Hepatic	2 (7)	1 (3)	2 (1)	1 (1)
Renal	1 (3)	1 (3)	1 (1)	1 (1)
Haemorrhage	1 (3)	0	1 (1)	0
Nausea, vomiting	8 (27)	1 (3)	16 (15)	1 (1)

. There was one episode of febrile neutropenia lasting 4 days. Grade 4 thrombocytopenia requiring transfusion occurred in two patients. Seven whole blood transfusions were given during the course of the study. Growth factor support was required in two patients. There was no episode of bleeding.

## DISCUSSION

In this study, we have achieved an RR of 36.6% (95% CI 34–40%) in 30 evaluable patients, with median response duration of CR/PR being 58/20 weeks. With an additional 23.3% patients experiencing stable disease, chemotherapy resulted in abrogation of progression in 60% of the patients.

Drugs including 5-flourouracil, mitomycin C, cisplatin, methotrexate, etoposide, doxorubicin, nitrosoureas, paclitaxel, and irinotecan have been tested either as single agents or in combination, without appreciable efficacy in the treatment of advanced gall bladder cancer. Clinical trials in gall bladder and hepatobiliary cancers have suffered due to the rarity of occurrence. The small number of patients in these studies does not allow any conclusive evidence to be drawn. Carcinomas of the biliary tract and gall bladder may behave differently with respect to the biological behaviour and the response to treatment. A prospective phase III study from Japan has shown a benefit of systemic chemotherapy in patients with gall bladder cancer undergoing noncurative resections (Takada *et al*, 2002). A similar benefit was not seen in other biliary tract cancers.

Since the commencement of this trial, a number of reports about the efficacy of chemotherapy in biliary tract cancer have appeared in the literature. In phase II trials evaluating single-agent activity of gemcitabine in over 160 patients with advanced biliary tract cancer, objective RR up to 60% and abrogation of PD (CR+PR+SD) in 50–93% of patients have been reported (Metzger *et al*, 1998; Kubicka *et al*, 1999; Valencak *et al*, 1999; Dobrila *et al*, 2000; Arroyo *et al*, 2001; Gebbia *et al*, 2001; Penz *et al*, 2001).

Combination chemotherapy of gemcitabine with other agents in phase II trials involving approximately 130 patients has shown responses ranging from 9 to 53%, with a tolerable toxicity profile (Andre *et al*, 2001; Carraro *et al*, 2001; Gebbia *et al*, 2001; Kuhn *et al*, 2001; Kornek *et al*, 2002; Scheithauer, 2002; Murad *et al*, 2003). Overall survival in these studies ranged from 6.3 to 16 months. Most of these studies have included all biliary tract cancers. The preliminary results of our study had also confirmed these findings (Doval *et al*, 2001, 2002). A recent phase II study (Malik *et al*, 2003) using the combination of gemcitabine and cisplatin in advanced gall bladder cancer has reported high activity (64% RR) with a tolerable toxicity profile. The encouraging results of this combination in the present study, along with the reports in other phase II trials, suggest that gemcitabine may have an active role in the management of gall bladder cancer. The present data and literature review, however, do not address the question whether this combination is superior or equivalent to single-agent gemcitabine.

Further research in this area should be directed to define the best cytotoxic agent for combination with gemcitabine, or altering the dose intensity or route of administration in advanced gall bladder cancer. A larger trial of gemcitabine alone *vs* combination with cisplatin compared with 5FU alone, needs to be conducted. Also, the role of gemcitabine/cisplatin combination in the adjuvant setting in suboptimally resected patients should be further pursued.
